# Does Compression Sensory Axonopathy in the Proximal Tibia Contribute to Noncontact Anterior Cruciate Ligament Injury in a Causative Way?—A New Theory for the Injury Mechanism

**DOI:** 10.3390/life11050443

**Published:** 2021-05-14

**Authors:** Balázs Sonkodi, Rita Bardoni, László Hangody, Zsolt Radák, István Berkes

**Affiliations:** 1 Department of Health Sciences and Sport Medicine, University of Physical Education, 1123 Budapest, Hungary; berkesdr@gmail.com; 2 Department of Biomedical, Metabolic and Neural Sciences, University of Modena and Reggio Emilia, 41125 Modena, Italy; rita.bardoni@unimore.it; 3 Department of Traumatology, Semmelweis University, 1145 Budapest, Hungary; laszlohangody@gmail.com; 4 Research Center for Molecular Exercise Science, University of Physical Education, 1123 Budapest, Hungary; radak@tf.hu

**Keywords:** non-contact ACL injury, proximal tibia, proprioception, acute compression axonopathy, NO, prostaglandin E2, glutamate, NMDA receptor, NGF-TrkA axis

## Abstract

Anterior cruciate ligament injury occurs when the ligament fibers are stretched, partially torn, or completely torn. The authors propose a new injury mechanism for non-contact anterior cruciate ligament injury of the knee. Accordingly, non-contact anterior cruciate ligament injury could not happen without the acute compression microinjury of the entrapped peripheral proprioceptive sensory axons of the proximal tibia. This would occur under an acute stress response when concomitant microcracks-fractures in the proximal tibia evolve due to the same excessive and repetitive compression forces. The primary damage may occur during eccentric contractions of the acceleration and deceleration moments of strenuous or unaccustomed fatiguing exercise bouts. This primary damage is suggested to be an acute compression/crush axonopathy of the proprioceptive sensory neurons in the proximal tibia. As a result, impaired proprioception could lead to injury of the anterior cruciate ligament as a secondary damage, which is suggested to occur during the deceleration phase. Elevated prostaglandin E2, nitric oxide and glutamate may have a critical neuro-modulatory role in the damage signaling in this dichotomous neuronal injury hypothesis that could lead to mechano-energetic failure, lesion and a cascade of inflammatory events. The presynaptic modulation of the primary sensory axons by the fatigued and microdamaged proprioceptive sensory fibers in the proximal tibia induces the activation of N-methyl-D-aspartate receptors in the dorsal horn of the spinal cord, through a process that could have long term relevance due to its contribution to synaptic plasticity. Luteinizing hormone, through interleukin-1β, stimulates the nerve growth factor-tropomyosin receptor kinase A axis in the ovarian cells and promotes tropomyosin receptor kinase A and nerve growth factor gene expression and prostaglandin E2 release. This luteinizing hormone induced mechanism could further elevate prostaglandin E2 in excess of the levels generated by osteocytes, due to mechanical stress during strenuous athletic moments in the pre-ovulatory phase. This may explain why non-contact anterior cruciate ligament injury is at least three-times more prevalent among female athletes.

## 1. Introduction

Anterior cruciate ligament (ACL) injury occurs when the ligament fibers are stretched, partially torn, or completely torn [1]. The average annual increase of ACL injury has been shown to be 1.3% over the 16 years of surveillance data of collegiate athletes [2]. The vast majority of ACL injury occurs on a noncontact basis, comprising 70–84% [2,3,4,5,6,7,8].

The exact mechanism of noncontact ACL (NC-ACL) injury is not completely understood. Boden et al. [9] examined the current theories of NC-ACL injuries including: impingement in the intercondylar notch [10], quadriceps contraction [11], quadriceps-hamstring imbalance and axial compressive forces on the lateral aspect of the joint [12,13]. Female athletes have an almost three times higher incidence of occurrence than male athletes [14,15]. Boden et al. [9] also examined the proposed factors that could increase the risk of NC-ACL injury in female athletes, such as increased knee valgus or abduction moments, generalized joint laxity [10], knee recurvatum [7], ACL diameter [16] and hormonal effects of estrogen on the ACL [16].

Anatomical and sex differences are implicated in the risk of ACL injury [17,18,19], but impaired neuromuscular control has also been identified as a risk factor, including decreased neurocognitive function [20], increased trunk displacement after sudden force release [21], and the weakened neuromuscular control of the core and hip musculature [17,22].

The authors propose that the initial cause of NC-ACL injury is an acute damaging compression injury of the proprioceptive sensory axons in the proximal tibia. The axial impulsive force theory implied two compression forces that are attributed to NC-ACL injury, namely the compressive valgus force due to leg buckling and the compressive anterior force due to quadriceps contraction [9]. Boden et al. also attributed the cause to the combination of forces and suspected an external impulsive axial force as the primary source [9]. The superposition principles [23] of physics state that the superposition of compression waves results in an even higher compression force. The authors are suggesting that the superposition of burst compression forces could cause the initial neuronal microdamage within the proximal tibia, which suggests that an acute compression axonopathy could prevail due to this extreme force. Most NC-ACL injuries happen when “foot strike with the knee close to full extension” [24]. This is the provocative position when superposition of damaging compression forces is maximized.

The authors of this paper emphasize the importance of the large fiber proprioceptive sensory neurons in the periosteum and in the proximal tibia. They believe that the compressive mechano-energetic lesion of these proprioceptive axons could precede the NC-ACL injury. In fact, their theory entails that ACL injury cannot happen on a noncontact basis without the preceding compressive impairment of these proprioceptive sensory neurons. Delayed onset muscle soreness (DOMS) consists of two damaging phases [25,26,27] where the initial damaging superposition of compression forces are proposed to microdamage the proprioceptive terminals in the muscle spindle. Accordingly, the current authors suggest that NC-ACL injury is also comprised of two phases. It is noteworthy that the similarity of the innervation of periosteal bone compartments and the muscle spindles has been emphasized [27]. The primary phase of the NC-ACL is suggested to be a burst compression or crush axonopathy of the fastest conducting proprioceptive sensory axons in the Aβ range of the proximal tibia. The secondary phase occurs when the subsequent superposition of compression forces results in the injury of the ACL due to already impaired proprioception.

Athletes seem to be at the greatest risk for NC-ACL injury towards the end of half-time, at the finishing of games and at season end [28]. Furthermore, we could learn from reported ski accidents that close to one third of ACL injuries happen at the first day of skiing and 57% within the first two days [29]. Not to mention the ‘one last run’ and ‘last hour’ ski related ACL injuries. The above mentioned implies that unaccustomed and strenuous fatiguing eccentric exercise, e.g., skiing is a typical one, could increase the risk of NC-ACL injury, as we could see in DOMS inducing exercises. Accordingly, the current authors are proposing that NC-ACL injuries happen under a cognitive demand derived acute stress reaction (ASR) when insufficient force production is unacceptable in unaccustomed and strenuous fatiguing eccentric exercise moments [27,30].

## 2. Hypothesis: NC-ACL Injury Is Caused by Acute Compression Axonopathy Followed by a Harsher Secondary Damage Including the Injury of the ACL

The authors of this paper suspect that the mechanism of NC-ACL injury is divided into two phases, where the primary damage is followed by an even harsher secondary damage, like in DOMS [25,26,27]. They are proposing that the critical damage of the first phase in NC-ACL injury is due to large fiber sensory axonopathy caused by superposition of compression forces. NC-ACL injury would not happen without the compression nerve injury of these sensory axons.

Olsen et al. [24] concluded from their video analysis that the ACL injury of female handball players happened generally in two scenarios, “a plant-and-cut faking movement or a 1-leg landing from a jump shot with the same pattern”. The injury mechanism was attributed to “a forceful valgus collapse from position with the knee close to full extension combined with slight rotation of the tibia. The foot was solidly planted on the ground mostly outside the knee.” These situations are similar to injuries found with soccer players, where NC-ACL injuries occur “during pressing followed by kicking and heading”. Only knee valgus was observed very often, while valgus collapse was rare among soccer players [31].

In another observational study, ACL injury was attributed to “change of direction or cutting maneuvers combined with deceleration, landing from jump in or near full extension, pivoting with near fully extended knee and a planted foot”. In most NC-ACL injury cases, the mechanism could be described by “a deceleration task with high knee internal extension torque combined with dynamic valgus rotation with the body weight shifted over the injured leg and the plantar surface of the foot fixed flat on the playing surface” [32].

It is often cited that ACL injuries are happening in maneuvers that have been performed by athletes on numerous occasions [33,34]. The authors argue that two additional aspects of the injury mechanism should be considered in addition to earlier theories: the situational difference under enhanced cognitive demand induced ASR and the deceleration being preceded by acceleration and deceleration moments when the acute axonopathy could be already initiated.

### 2.1. Primary Damage Phase: Acute Compression Axonopathy Caused by Superposition of Compression under an Acute Stress Reaction

The authors of this paper suggest that during unaccustomed or strenuous exercise, activity-derived stress could be enhanced by increased cognitive demand. It is demonstrated that dual-task (cognitive and gait functions are used at the same time) could induce a more significant stress in the form of an acute stress response and could impair proprioception leading to falls, compared to single task in the elderly [35]. The author of this paper further suggest that the same result could occur in unaccustomed or strenuous athletic moments when force production is depleted, but cognitive demand derived ASR is induced in order to sustain athletic performance [27,30]. It is important to note, that this translation applies only to the relationship between cognitive demand induced ASR and proprioception in force production depleted dual-task moments, and not on the fall of elderly which is a multifactorial construct. Under these moments cognitive and sensimotor neuro-energetics have resource limitations in the modulation process. As a result, the neuromuscular control of upper body rotations, hip/knee and ankle is reduced with the increase in the complexity of a dual task. Therefore, postural control is decreased with dual-task difficulty enhancement [36].

Part of cognitive demand, as players reported, are “being out of balance, being pushed or held by an another opponent, trying to evade a collision with the opponent, and having an unusually wide foot position” [24]. All these circumstances should be calculated into the planning of complex task execution and exert a significant loading factor on proprioception in an already depleted situation. Even though the basic maneuver patterns seem to be those used by professional athletes, the enhanced cognitive load implies the inclusion of additional stress and fear factors. Furthermore, the stress loading is already increased by the fact that 75% of the injuries happen during games [33], not to mention that the greatest risk for NC-ACL injury is towards the end of half-time, at the finishing of games and at season end [28] when the stakes are higher.

Hereby, we should consider strenuous competitive moments of games and practices that induce cognitive demand substantially in order to execute complex tasks. Motor planning and working memory are sharing the same limited neuro-energetic resources [35]. Thus, the abrupt reallocation of these scarce neuro-energetic resources could be impaired when task complexity increases in unaccustomed or strenuous competitive moments. The involved part of the nervous system which has the highest energy demand will be affected negatively first in this process, and that part is suggested to be proprioception.

Charles “Buz” Swanik emphasized the brain’s role in NC-ACL injuries and devoted “inattentional blindness” as an exposing factor for capsuloligamentous structures to failure. Inattentional blindness occurs when one fails to perceive unexpected objects [37]. John A. Spinks et al. [38] demonstrated that loading the working memory with cognitive task impaired automatic processing in response to distracting objects. Furthermore, Rees et al. [39] showed that visual perception of objects largely depends on attention. Inattentional blindness is not due to the absence of cognitive processing, but rather relates to how one consciously fails to perceive objects. The authors of this paper propose that in strenuous or unaccustomed exercise moments, when ASR is induced in order to satisfy cognitive demand, difficult task execution is attentionally so demanding that scarce neuro-energetic resources could induce more inattentional blindness moments. Therefore, inattentional blindness could disturb proprioceptive integration on the supraspinal level of difficult task execution. It is important to note that inattentional blindness is likely not the cause, but a neuroenergetic saving effect under ASR that could disturb the proprioception. For example, inattentional blindness alone would not explain the significantly higher prevalence of female athletes with NC-ACL injury [14,15].

It is important to note that bone-derived signaling seems to be essential in strenuous athletic moment derived ASR initiation. Berger et al. [40] demonstrated that the quick outflow of osteocalcin inhibits the parasympathetic neurons and facilitates the unfolding of an ASR. Exercise under ASR has two dimensions: “heat of battle” response and “fight or flight” response. The “fight or flight” response dampens the feedback control of muscle length by increasing sympathetic loading [41,42,43,44]. The trading of fine movements for low feedback control is beneficial in ASR [41], because neuro-energetic resources could be mobilized toward the fight or flight response. The “heat of battle” response is facilitated by sympathetic nervous system activity that suppresses pain by descending inhibition of nociception in the spinal cord [45] and by the faster conduction velocity of the non-nociceptive Type Ia sensory fibers that indirectly inhibit the effects of nociceptive Type II sensory fibers [27]. Therefore, the difficult task with higher force generation could be executed under ASR without the immediate limitation of pain.

Difficult task execution under enhanced cognitive demand, with the assistance of ASR, increases the force generation of eccentric contractions in acceleration and deceleration movements. Eccentric contractions have the characteristics of absorbing energy from an external load [46], supporting the body against gravity, absorbing shock, and storing recoil energy for accelerating contractions [25,47]. Eccentric contractions employ higher cortical excitation and lower motor unit discharge [25,48], providing the base for integrating task execution according to cognitive demand. As a result, the accelerating or decelerating eccentric contractions with high force generation impose substantial compression on the knee joint and even more specifically on the tibia.

The quadriceps is the strongest weight-bearing muscle in this mechanism and its contraction substantially enhances compression on the tibiofemoral joint and the tibia [49], not to mention valgus bending and tibia rotation that further enhance shear force. The other compressing force affecting the tibiofemoral joint and the tibia comes from ground reaction forces (GFR), that is the force applied by the ground as an answer to the forces a body applies on it [50], especially when it is not absorbed adequately by the lower limbs [9]. We suggest that the superposition of these compression forces is microcracking the proximal tibia even prior to NC-ACL injury.

Bone bruises are manifest after NC-ACL injury. The prevalence of bone bruises is increasing and could be up to 98% (78% in the last 10 years). The rapid development of MRI technology and the resultant more precise diagnostics are behind the increasing trend [51]. It is noteworthy that Meyer et al. [52] could also demonstrate, with micro-computed tomography scans, that microcracks were present at the subchondral bone due to valgus bending. Furthermore, the injury pattern did not change when additional tibiofemoral compression was applied, but the osteochondral damage was even more significant. The authors of this paper are suggesting here that microcracks are present prior to NC-ACL injury. The large fiber axons of the fastest conducting sensory neurons in the Aβ range of the tibia are also being microdamaged concomitantly with microcracks prior to NC-ACL injury. The primary injury as an axonopathy could happen already at the preceding accelerating and decelerating moments of strenuous or unaccustomed athletic exercise bouts with an abrupt, excessive compression force (including shear force) that is causing a nerve compression or crush injury. Therefore, an acute sensory axonopathy could precede NC-ACL injury, by happening before the decelerating moment. According to this theory, the entrapped proprioceptive sensory axons are already impaired in the decelerating moment, otherwise ACL injury would not happen on a noncontact basis.

Zazulak et al. [51], in a three-year follow-up prospective biomedical–epidemiological study, mentioned the deficient proprioception and the resultant excessive lateral trunk displacement in female athletes as a predictor of NC-ACL injury. In a separate survey, coaches also noticed the impaired proprioception of NC-ACL in injured athletes [24]. Pain from peripheral sensory nerve injury could stay silent under an ASR; therefore, only the impaired proprioception could be indicative of the possible preceding microinjury of the sensory nerves, namely the fastest conducting sensory axons in the Aβ range of the proximal tibia. Furthermore, the microcracks in the epiphysis of the tibia caused by superposition of compression (including valgus bending) could be another important sign of preceding microdamage before the actual NC-ACL injury. We argue that the concomitant burst compression or crush of the axons of the fastest conducting sensory neurons of the periosteum and epiphysis by the same damaging force could contribute to the impairment of proprioception. It should not be forgotten that the proposed signaling, that highlights excessive eccentric force generation by an ASR [27,30], is bone derived as well, and is demonstrated in the form of immediate release of circulating osteocalcin [40].

**Figure 1:**
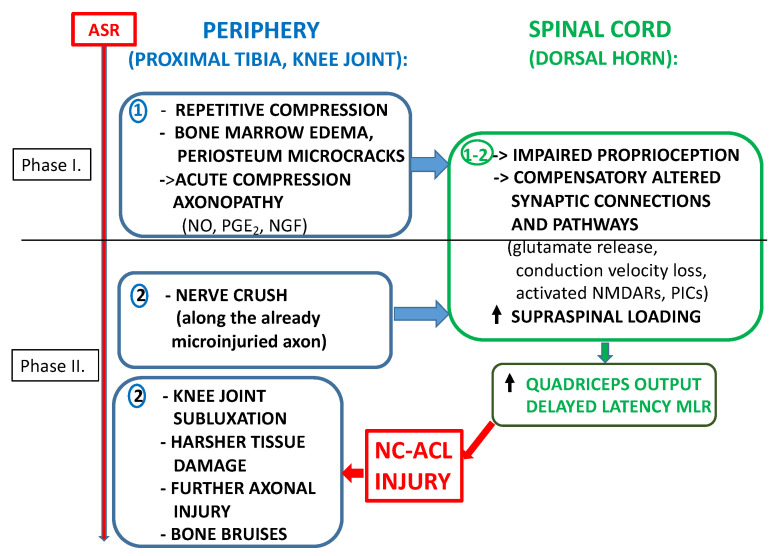
Schematic representation of the peripheral and central mechanisms involved in Non-Contact Anterior Cruciate Ligament (NC-ACL) Injury at the periphery NC-ACL injury is comprised of 2 phases: Phase I: The acute compression axonopathy of proprioceptive sensory fibers in the proximal tibia. Phase II: Nerve crush along the already microinjuried axon. A more extensive secondary damage occurs, including further tissue damage and bone bruises. This is the phase when the actual NC-ACL injury happens. In the spinal cord, the peripheral alterations cause changes in the functional properties of sensory afferent fibers: a: The hyperexcited, microdamaged large encapsulated Aβ fibers exert less presynaptic inhibition, or even presynaptic facilitation, on proprioceptive Type I fibers, by activating presynaptic NMDA receptors. This causes some of the monosynaptic static encoding of stretch reflex to be altered to polysynaptic pathways and it is the hypothetical basis of the delayed latency of the medium latency response (MLR) and impaired proprioception. Glutamate spillover from Type I fiber central terminals could in turn induce presynaptic facilitation on Type II fiber terminals, increasing the release of glutamate. This leads to the excitation of spinal cord motoneurons, evoking persistent inward currents (PICs) and enhancing the quadriceps output, that could be further increased by decreased GABAergic inhibition. b: The conduction velocity of the hyperexcited, microdamaged large encapsulated Aβ fibers decreases as the parasympathetic withdrawal of ASR evades. This reduces the presynaptic inhibition on pain fibers (Aδ and C), opening the gate for pain transmission.

In summary, the authors are suggesting that the primary damage phase could happen in the preceding accelerating and decelerating moments due to the acute compression axonopathy of the entrapped proprioceptive sensory neurons of the proximal tibia. The damaging compression is caused by burst superposition of compression forces, including shear force (see Phase I in Figure 1).

### 2.2. Secondary Damage Phase: Includes the NC-ACL Injury Due to Impaired Proprioception

Impaired proprioception increases trunk displacement [21] and weakens core and hip muscles [17,22,53]. The result is exaggerated contractions in the quadriceps and weakened contractions in the hamstring and in those muscles (hip and soleus) that are responsible for absorbing the excess force. The proprioceptively impaired muscles are weakened against gravity and, due to inertness, the body weight further loads the ACL at near full extension of the knee. We suggest that the exaggerated contraction of quadriceps muscles is caused by the reflexive stabilization of the postural control, induced by the already microinjuried proprioception [30]. Paradoxically, the exaggerated quadriceps output further increase the load on the ACL [9,11]. Under these circumstances a subsequent burst superposition of compression force could reinjure the already impaired proprioceptive sensory nerves and the ACL injury could prevail as an even harsher secondary damage. The suggested mechanism is in line with the theory of Upton and McComas who introduced the term “double crush syndrome” in 1973, when they hypothesized that the same functionally impaired axons, due to compression, are more prone to axonal damage at another site [54]. Double crush syndrome also means that compression at more than one location along a peripheral axon could “synergistically increase symptom intensity” [55]. The current authors are proposing that the second hit of the microdamaged proprioceptive axon will further increase the impaired proprioceptive symptoms and lead to the injury of the ACL.

The systemic review of Filardo et al. [51] cited studies that showed a correlation between the extent of bone bruise and higher pain and laxity after ACL injury. These findings suggest impairment of proprioceptive sensory nerves and the excitement of nociceptive sensory nerves after injury. Boden et al. demonstrated, using a video-based analysis, that those who experienced NC-ACL injury landed on the ground with the hindfoot or with the foot flat, while those who did not suffer ACL injury landed on the front of the foot. Furthermore, the injured athletes had significantly less ankle plantar flexion and reached the flat footed position up to 50% sooner [9]. These findings indicate that proprioception could already be impaired preceding the injury of the ACL, and the anti-gravity protection of muscles are deficient. The authors of the present paper further propose that the significantly decreased landing time and arriving on hindfoot or flat foot is also the result of impaired proprioception. Therefore, the soleus muscle, the hamstrings, and the hip will not be able to absorb the excessive compression force, and a secondary damage will prevail.

The landing or decelerating mechanism also involves the superposition of compression forces and the anatomical predisposition which, eventually, leads to valgus bending, valgus collapse, tibia torsion or a combination of these. Boden et al. [9] demonstrated this anatomical predisposition and found that the ACL injury provocative position is also the one in which bone bruises occur. The superposition of compression forces comes from the impairment of weight-bearing and from GFR due to the deficiency of proprioception and will eventually result in tibia torsion, valgus bending or valgus collapse. Therefore, the secondary, more severe damage could be the result of an enhanced exposure to inertness due to the impaired proprioceptive protection and the injury of the ACL. Accordingly, the compressive tissue damage is even harsher in the secondary damage phase than in the primary one (see Phase II in Figure 1). Recent findings of Grassi et al. [56] are in line with this hypothesis since they concluded that bone bruises are in a subluxated position, reflecting a late phase of ACL injury and not the phase that is leading to ACL injury.

The combination of a constrained medial compartment and a relatively loose lateral compartment may allow the lateral tibial plateau to shift anteriorly with internal rotation, which can dramatically increase the anterior shear force and the strain on the ACL [57].

In summary, the storing of recoil energy from superposition of compression forces by eccentric contractions [25,47] in the accelerating phase backfires in the decelerating phase, because the excess energy will be even more damaging, due to acute compression axonopathy-induced-impaired proprioception.

## 3. Impairment of Proprioception

The handball coaches of ACL injured Norwegian athletes observed dysfunctional proprioception in the form of lost balance, coordination, and movement due to some form of perturbation [24]. These observations could suggest that the impairment of proprioception could precede the ACL injuring moment.

Dhaher et al. [58] highlighted that the source of joint stability could be represented by bone/cartilaginous contact forces [59], ligament and capsule stiffness [60,61], intrinsic stiffness of active muscles [62], and reflexively mediated muscle stiffness [63]. Daher et al. [58] also demonstrated that the stimulation of the afferents of the periarticular tissue enhanced the joint adduction-abduction stiffness that helps the knee joint in stabilization. If we look at the joint instability sources from the angle of proprioceptive afferents, we could then narrow the sources to muscle spindle, ligament/capsule and bone. Dhaher et al. [64] excluded the muscle spindle origin and suggested the knee capsule. The current authors are proposing that in perturbation induced moments the ligament/capsule proprioceptors modulate the static component of the muscle spindle encoding, as described by Dhaher et al. [64]. However, the authors argue whether this protective reflexive mechanism alone could lead to ACL injury on a non-contact basis. It is rather suggested that this perturbation induced reflex mechanism is a preprogrammed postural control adaptation that provides enhanced postural stabilization.

The authors of this paper theorize that there is no such magnitude of burst axial compression force on a non-contact basis that could microdamage the proprioceptive sensory nerves of the knee capsules due to the soft and more resilient tissue characteristics. Nevertheless, the possibility of the knee capsular contribution cannot be excluded. On the contrary, long bones, namely the tibia, are responsible for bearing the burst superposition of compression forces with a more rigid structure. This stress could cause microcracks and edema in the proximal tibia, that could make the fatigued proprioceptive sensory neurons more prone to compression or crush injury due to entrapment exposedness. Furthermore, these proprioceptive sensory neurons are larger in diameter, and intraneural edema could possibly occur under fatiguing conditions in such a stressful bony environment.

The saphenous nerve is often considered as a cutaneous sensory nerve and it has an infrapatellar branch [65]. Clendenen and Whalen [66] showed that the saphenous nerve innervates not only the skin, but the periosteum of the medial ankle and the joint capsule as well. Based on this finding, we hypothesized that the infrapatellar branch of the saphenous nerve might as well contribute to the innervation of the periosteum of the proximal tibia and knee joint capsule. In addition, the periosteal and joint capsule contribution at the medial ankle could go through the same injury mechanism potentially causing non-contact medial ankle sprain. The saphenous nerve is exposed to passive stretch when the knee is under extension or flexion, externally rotated and under valgus stress. The saphenous nerve is also often exposed to irritation and compression leading to saphenous neuritis. It is indicative that saphenous neuritis could cause medial meniscal tear or osteoarthritis. Furthermore, there is also evidence that osteochondroma or other soft-tissue and bony tumors could cause compression saphenous neuritis at the proximal tibia [65]. These pathological findings seem to support the current hypothesis that the edematous proximal tibia could lead to compression axonopathy. Edema of the proximal tibia is common even in asymptomatic athletes that could be due to repetitive microtrauma of jumping and running that is “transmitted through the meniscus, dissipated by the cartilage, and eventually absorbed into the bone” [67]. Major and Helms also found that 35% of examined collegiate basketball players had small joint effusion, and 41% had bone marrow edema [67]. Notably, arthrogenic muscle inhibition, which is a significant quadriceps muscle weakness after injury, surgery or pathology affecting the knee, could be evoked by experimental knee joint effusion. Rice et al. [68] also showed there was no supraspinal contribution to quadriceps arthrogenic muscle inhibition, but an increase in quadriceps corticospinal excitability, as it was found earlier. This paradoxical increase of corticospinal excitability was partially attributed by Rice et al. to a decrease in gamma-aminobutyric acid (GABA)-ergic inhibition within the motor cortex [68]. The current authors suggest that bone marrow edema of the proximal tibia and, less likely, the joint effusion of the knee joint could compressively microdamage fatigued large and/or small sensory fibers that contribute to proprioception, initiating the exaggerated output of the quadriceps and the decreased GABA-ergic inhibition under an ASR in the NC-ACL injury mechanism.

Proprioception is autonomous without consciousness, and as a result, the informativeness with movements is decreasing [69,70,71]. The purpose of practice for athletes is to overlearn task execution. Proprioceptive feedback is extensively used in the learning stage of task practice. Once the practiced task is overlearned, it could then be executed autonomously without increasing the loading of proprioception. The situation is significantly different when the practiced task should be performed with further cognitive demand, because the matching could be accomplished only by further proprioceptive input [71,72]. Acute stress response (ASR) is induced when muscle is fatiguing, force generation is inappropriate and energy is being depleted in a strenuous or unaccustomed athletic moment. We suggest that proprioception could be impaired under these ASR moments on a supraspinal, spinal or even peripheral level [1,27,73]. This abrupt neuro-energetic depletion of supraspinal and spinal proprioceptive energy could make the peripheral proprioception more prone to oxidative stress in a situation when it is already under mechano- and energetic duress and this could lead to impaired peripheral neural control. Haid and Federolf [36] have demonstrated this phenomenon by increasing the difficulty of dual task, which results in decreased postural control. The mediolateral ankle sway was increased, pointing toward diminished control [36]. Saxton et al. showed in eccentric exercise that both joint position and force sense are impaired [71,73,74], but in other studies, it was found that the tendon organs and the muscle spindles were not to blame for this impairment [71,75,76]. Notably, the current hypothesis implies that the exaggerated output of the quadriceps with diminished neural control is only temporary under an ASR in order to fulfill the cognitive demand derived exercise, thereafter arthrogenic muscle inhibition prevails when ASR subsides.

Excitation of the ACL during exercise activity completely blocks muscle activity and provides a subjective “giving way” feeling [77]. Furthermore, Di Fabio et al. [78] showed that in case of ACL injury, an induced hamstring activity and resultant capsular-hamstring reflex is integrated into the preprogrammed postural synergy in order to compensate for joint laxity. The activation of the contralateral hamstring was not associated with postural response, while the injured extremity showed a postural response coupled with hamstring activation. The activation of the hamstring is usually not involved in backward perturbations [78]. Only the second component of the capsular-hamstring reflex, the medium-latency reflex (neither short-, nor long-), contributes significantly to the sensorimotor function of the knee joint [79]. The delayed latency of the medium latency stretch reflex (MLR) of vasti muscles is also implicated in the valgus collapse phase of the NC-ACL injury mechanism, suggesting the contribution of non-muscular mechanoreceptors [64]. We suggest that the delayed latency of MLR is indicative of impaired proprioception and it could be due to exchange of monosynaptic static encoding of the stretch reflex to polysynaptic ones in order to enhance postural control [30]. It is noteworthy that this exchange means not only enhanced proprioceptive integrational and compensatory loading on spinal level, but evidently on supraspinal level as well, where visual and sensimotor integration takes place. It is indicative that after ACL injury, when proprioceptive impairment is evident, the frontal cortex and visual–spatial brain region activity increases, and this increased load reduces postural stability [80,81]. Furthermore, the delayed latency of MLR could also mean that the strained ACL is overexposed time-wise to straining forces at the most provocative position, which is the knee at close to full extension and a delay in spatial encoding from the periphery. This is the basis why non-contact injury mechanism can be attributed to sensimotor prediction errors [81,82,83] and the video-analysis findings of Boden et al. [9]. It has been already proposed by Proske and Gandevia [71] that the damaging eccentric exercise is to blame for the impairment of proprioception. We suggest that the size of an abrupt eccentric exercise could lead to compression or crush microdamage of the fatigued proprioceptive sensory fibers in the periosteum, epiphysis, or/and the subchondral region of the proximal tibia. As a result, the proprioceptive contribution is already impaired in the secondary damage phase of NC-ACL injury when the actual injury of the ACL is suspected.

## 4. Innervation and Neural Control of the Proximal Tibia and Its Relevance in NC-ACL Injury

Femur and tibia have the highest weight-bearing function among bones. They are wrapped by periosteum [84]. The periosteal bone compartments have similar neuroanatomical and functional features like the muscle spindles [27]. The periosteum of the long bones is densely innervated with sensory neurons as is the case in the surrounding synovial membranes and also in the subchondral bones [85,86,87] of the knee-joint. The periosteum is innervated by a ‘neural net’ of Aδ and C sensory fibers in order to signal stretch pressure in the form of mechanical injury or distortion of the bones [85,88]. There are few non-nociceptive, low threshold Aβ large fibers with encapsulated endings in the periosteum [85]. Most of the thinly myelinated Aδ fibers are peptide rich, calcitonin gene-related peptide (CGRP) positive, tropomyosin receptor kinase (TrkA) positive, high-threshold sensory fibers, which respond to nerve growth factor (NGF) and are considered to be nociceptive. The rest of the thinly myelinated Aδ fibers are TrkA negative, non-nociceptive, and low threshold neurons. The majority of the nociceptive unmyelinated C fibers are high-threshold peptide rich, CGRP positive, Substance P positive and TrkA positive, and respond to NGF. A very small portion of the nociceptor unmyelinated C fibers is TrkA negative, peptide poor, and reacts to glial cell line-derived neurotrophic factor (GDNF) [85,89,90,91]. The nociceptive C fibers have the smallest diameter axons with the slowest conduction velocity. Therefore, they are believed to be the mediators of ‘slow pain’. The thinly myelinated nociceptive Aδ fibers have larger diameters with faster conduction velocity, thus they are suggested to convey ‘fast pain’. Aβ large fibers have the fastest conduction velocity and they are usually transmitting mechanoceptive and proprioceptive impulses [91,92]. Many of these nerves penetrate the cortical bone alongside the ligamentous Sharpey’s fibers, suggesting further sensory perception within the cortical bone [93]. The afferent sensory nerves of the tibial epiphysis exit through the intercondylar foramina [84] in close vicinity to the cruciate ligaments [94].

We propose that the large encapsulated sensory fibers in the periosteum are responsible for mechano- and proprioception, because large fibers have a higher conduction velocity and highly energized terminal compartments. It should not be forgotten that osteocytes, which consist of 90–95% of bone cells, are mechano-receptive to mechanical stresses as well, especially when it comes to shear stress [95]. The mechanical stress activated integrin could induce osteocytes to release prostaglandin E2 (PGE2) into the extracellular space [96]. Furthermore, osteocytes could function as an endocrine organ for even muscle cells with this PGE2 release mechanism [96]. Accordingly, we suggest that the osteocytes could function as neuromodulators: the increased level of PGE2 could also excite the nerve endings of those sensory neurons, innervating the periosteum and the epiphysis, that are conducting in the Aβ range. There are two types of sensory neurons in this range: the non-nociceptive encapsulated large fiber sensory neurons in the periosteum of the tibia [92,97] and the nociceptive stretch reactive ones with free fiber endings [92,98]. We propose that only these Aβ fiber have the conduction velocity range and features to contribute to proprioception and to related impairment signaling.

Proprioceptors are guarding the spine on a continuous basis in order the prevent pathology to happen, and there is an immediate response to maintain balance in case of a fractured bone [99]. Furthermore, Blecher et al. [100] demonstrated that muscle spindles and Golgi tendon organs contribute to spine realignment and fractured bone realignment. We suggest that the fastest conducting proprioceptive sensory neurons in the Aβ range of the epiphysis and periosteum of the tibia also serve this preventive stabilizing purpose when bone micro- or stress fracture happens, in accordance with the mechanosensors, like muscle spindles. Recent research in mice demonstrates that even spontaneous fracture repair is guided by monosynaptic stretch reflex circuitry with the active assistance of the muscles and the involvement of the proprioceptive system in a non-autonomous way [99]. Epiphyseal large fiber sensory neurons in the tibia are suggested to have an important role in the maintenance of bone structure [84] and most likely in spontaneous microfracture repair as well. We suspect that under ASR the proprioceptive sensory neurons could have an analog role in the maintenance of the periosteum and in the spontaneous stress fracture/microcrack repair as well.

The authors of this paper propose that NC-ACL injuries occur in a similar fashion as in vertebral compression fractures, which are secondary fractures. The primary fracture, called burst fracture, is caused by an abrupt axial impact which leads to biomechanical impairment and eventually to the secondary compression fracture [101]. In younger patients this bimodal mechanism has often been seen in speedboat vertebral fractures [102]. We translate these findings that the abrupt axial damaging load could induce compression or crush of the affected proprioceptive sensory axons with a resultant dysfunction of proprioception and more mediolateral sway in the joints, leading to dislocation. A secondary compression fracture occurs due to impaired proprioception induced inadequate postural control, inadequate shock absorption, inadequate anti-gravity protection and inertness. The recent findings of Grassi et al. seems to substantiate the secondary subluxation in NC-ACL injury [56].

Compression injury of peripheral nerves usually happens in locations where nerves travel through narrow anatomical pathways [103]. The periosteum, epiphysis and the subchondral region of the proximal tibia could be such environments under mechanical stress. Crush injuries are usually caused by an acute traumatic compression without the transection of the nerve [103]. Narrow and solid anatomical structures like bones could make proprioceptive sensory fibers more prone to nerve compression or crush injury, especially under fatiguing conditions.

According to the Gate Control Theory of Pain [104], the faster conducting non-nociceptive fibers (encapsulated large fibers) indirectly inhibit the nociceptive fibers (stretch reactive free fiber endings conducting in the Aβ range) by closing the gate to transmission of pain stimuli [105]. In the spinal cord, stimulation of Aβ fibers activate inhibitory interneurons: these, in turn, release GABA and/or glycine that inhibit both spinal cord interneurons (postsynaptic inhibition) and primary afferent terminals (presynaptic inhibition). In particular, presynaptic modulation of the proprioceptive input, together with postsynaptic mechanisms involving spinal interneurons, contributes to the control of spinal reflexes, such as those involved in the generation of AMI [68]. Presynaptic inhibition is largely due to the mechanism of “primary afferent depolarization”, primarily mediated by GABA_A_ receptors expressed on primary afferent terminals [106]. Beside GABA_A_, also glutamate α-amino-3-hydroxy-5-methyl-4-isoxazolepropionic acid (AMPA) and N-methyl-D-aspartate (NMDA) receptors, expressed on both large and small diameter afferent fibers, contribute to primary afferent depolarization and presynaptic modulation in the spinal cord. NMDA receptors, in particular, can modulate transmitter release by depolarizing the afferent fibers, by causing calcium influx into the terminal and by triggering downstream intracellular signaling cascades [107].

Since we are suggesting that the large fibers in the Aβ range could be microdamaged in the primary injury phase, large encapsulated fibers could decrease conduction velocity and therefore the nociceptive pain stimuli could arrive at the gate earlier as it is suggested in DOMS [27]. The authors of this paper are further proposing that conduction velocity loss of large encapsulated Aβ fibers are either abrupt or delayed. Pre- and postsynaptic inhibition of spinal nociceptive circuits, driven by large fibers in the Aβ range, could also be impaired, contributing to the opening of the gate for pain transmission [106,108]. Regardless, the pain sensation will not be experienced because this strenuous athletic moment is under an ASR.

It has been observed that after nerve crush the motoneurons gain supranormal output regardless of slower sensory input [104]. Dendrites of motoneurons could actively generate persistent inward currents (PIC) that could explain this unexpected motoneuron gain [109]. This phenomenon could explain the exaggerated output of the quadriceps muscles prior to and throughout the point of NC-ACL injury. The authors are suggesting that the compressive hyperexcitation by microinjury of the Aβ fiber in the proximal tibia will elicit a similar exchange of static sensory encoding on the segmental dorsal horn that is proposed in DOMS [30]. The hyperexcitation of Aβ fibers could determine a decrease of presynaptic inhibition of Type I fibers, or even their facilitation, by activating presynaptic glutamatergic receptors, such as NMDA receptors. The subsequent increase of glutamate release from Type I terminals could activate, by spillover, Type II sensory fibers, allowing the static encoding of these fibers to arrive earlier to the ‘gate’ than the bypassed static encoding of the Type Ia sensory fiber. Indeed, while the physiological function of presynaptic NMDA receptors in spinal cord is still controversial, a critical role of these receptors in potentiating glutamate release from primary afferents has been observed after peripheral nerve injury, when their function is increased by receptor phosphorylation and interaction with other membrane channels [110].

This exchange of static sensory encoding between Type II and Type Ia sensory fibers could induce PICs on the dendrites of motoneurons, like it is suggested in DOMS [30]. Beside activation of presynaptic NMDA receptors, the reduction of GABAergic inhibition in the spinal cord ventral horn could also contribute to the generation of PICs [111]. Dendritic PICs will enhance and amplify the synaptic activation of motoneurons mediated by glutamate, leading to an increase of action potential firing [109]. The motoneuron gain could serve the purpose of postural control stabilization, enhanced shock absorption and anti-gravity protection. This could also be translated to explain why the neuro-energetic expense of the PIC derived segmental proprioceptive overcompensation of microinjured sensory axons is so high that the proprioception of other areas suffers in this abrupt neuro-energetic resource reallocation process. This, in turn, could lead to further impairment of proprioception, loss of shock absorption, inadequate anti-gravity protection and eventually to a more damaging secondary injury. Noteworthy, the exaggerated quadriceps output, which is meant to be protective, paradoxically increases the risk of ACL injury [9,11], because it exerts additional compressive load on the proximal tibia and strain on the ACL.

## 5. TAD Like Degeneration and the Role of Nitric Oxide (NO) in the Axonopathy

Osteocytes release nitric oxide (NO) as key signaling due to mechanical stress and especially to shear force [95]. The released NO eventually leads to osteoblast stimulation [112,113]. It is evident that in vivo blocking of NO synthase (NOS) leads to impairment of mechanical load-induced bone formation and fracture repair [114,115]. Furthermore, upregulation of NOS is essential in both mechanical load-induced bone growth and fracture repair [112,114,116,117,118,119,120,121]. NO acts in a dose dependent and biphasic manner [115], as the sensory neurons often function in a biphasic manner in the skeletal and muscle systems. At low concentrations, NO maintains bone homeostasis by stimulating osteoblasts and osteocytes and controlling osteoclast-mediated bone resorption [115]. On the contrary, at high concentrations, NO might induce bone loss [122]. High concentrations of NO also induce demyelination in case of axonal injury and the damage is selective to the axons of the sensory neurons in the form of a Wallerian-like degeneration [123]. Nonetheless, we propose that this type of NO induced sensory neuronal degeneration happens mostly in the more severe secondary damage phase of the NC-ACL injury, when extensive nerve crush injury could prevail.

The primary injury is suggested to be an axial burst loading of the knee joint due to superposition of compression, including shear forces in the periosteum, epiphysis or/and the subchondral region of the proximal tibia under strenuous athletic moments. As a result, we are suggesting two possible sensory nerve injury mechanisms in the primary damage phase: the primary burst microfracture could be so severe that the large fiber afferents with free nerve endings in Aβ range are crushed in the proximal tibia, and in this case the neuronal injury type will not be different from the one suggested in the secondary phase. The free radicals of NO could damage proteins, lipids, and peripheral nerve sensory axons [124], likely resulting in energetic failure [125] or even apoptosis [126]. Eventually, this could lead to increased nociception as well as the distal degeneration of nerve fibers [127]. The authors of this paper are suggesting that this sensory nerve injury mechanism could lead to valgus collapse.

In other cases, likely to be the most prevalent, the axial burst loading could microdamage the entrapped axon terminals of the encapsulated large fiber sensory neurons in the periosteum, analogously to what was proposed by the acute compression sensory axonopathy theory of DOMS [27]. In strenuous or unaccustomed athletic moments sophisticated task execution under ASR could impair proprioception due to abrupt reallocation of neuro-energetic resources [36]. As a result, the axon terminals of the encapsulated large fibers in the periosteum of the tibia could be vulnerable to free radical damage [128]. The encapsulated axon terminals are the compartments of the highest energetic demand [129] under this ASR-induced athletic moment, due to strenuous mechano-sensing excitation from superposition of compression including shear force. We propose the analog involvement of the mitochondrial electron transport chain-generated free radicals in the acute compression sensory axonopathy of large sensory fibers in the periosteum, like in DOMS. According to the acute compression sensory axonopathy theory of DOMS [27], the force due to the superposition of compression under ASR and cognitive demand could possibly cause a severe mechano-energetic insult on axon terminals. The energy supply of the mitochondria in the terminals of the large fiber sensory neurons of the periosteum are impaired in a way, which is similar to Bennett et al.’s hypothesis explaining terminal arbor degeneration (TAD) [127]. The authors of this paper are suggesting that this sensory axonal injury mechanism could lead to valgus bending.

The TAD mechanism could be evoked by axonopathy-causing chemotherapy agents, like paclitaxel and oxaliplatin [127,128]. Bennet et al. [127] showed in the paclitaxel model, that the appearance of symptoms was threshold driven by accumulating toxicity, and was also dosage dependent. Paclitaxel evoked TAD at low-dose thresholds, while at higher-dose caused axonal degeneration and apoptosis of the sensory neuron could happen at an even higher-dose threshold. The neuropathic symptoms induced by low-dose paclitaxel did not cause degeneration of the axon of the peripheral sensory nerve, but TAD lesion alone could have been sufficient to produce neuropathic symptoms [127]. We suggest the existence of a similar lesion on the nerve terminals of the encapsulated large fiber sensory neurons in the periosteum of the proximal tibia.

Oxaliplatin has neurotoxic effects in an acute and chronic manner [128]. In an eight year follow up study of oxaliplatin chemotherapy, periosteal apposition in the long bones was most apparent on the tibia. Furthermore, the periosteal apposition was also associated with noninflammatory arthritis of the large joints, such as knees. The clinical picture resembled to a primary disease, called hypertrophic osteoarthropathy (HOA) [130], in which circulating PGE2 level is significantly elevated [130,131,132].

In summary, we are suggesting that in the primary damage phase, the elevated NO and PGE2 from osteocytes could induce a burst sensory impairment on the compressed and crushed Aβ range large fibers in the periosteum, epiphysis or/and the subchondral region of the proximal tibia in unaccustomed and strenuous athletic moments. This acute sensory axonopathy leads to dysfunctional proprioception and eventually to the harsher secondary damage including the NC-ACL injury.

The excessively elevated PGE2 levels might explain the phenomenon that female athletes are more prone to ACL injuries in the pre-ovulatory phase of the menstrual cycle [32,133] when a marked elevation of estrogen is due to luteinizing hormone (LH) [32,133]. LH through interleukin-1β stimulates the NGF-TrkA axis in the ovarian cells and promotes TrkA and NGF gene expression and PGE2 release [134]. This mechanism could further elevate PGE2 in excess of the levels generated by osteocytes due to mechanical stress in strenuous athletic moments in the pre-ovulatory phase [96]. The pre-ovulatory transient surge of TrkA mRNA and NGF mRNA and concomitant PGE2 induced by LH is even more pronounced in puberty [134], which could explain why higher number of young female athletes suffer non-contact ACL injury [135,136]. Furthermore, it could explain why young female athletes have increased quadriceps activity and reduced hamstring activity [137], which are considered to be risks of NC-ACL injury.

## 6. ACL Injury Prevention: Neuromuscular and Proprioceptive Training

The usefulness of ACL injury prevention programs is emerging, although there is a long way ahead. Indeed, ample research work is needed to titrate these programs in order to enhance their effectiveness.

It appears to be evidence based that neuromuscular and proprioceptive training reduces ACL injury, although the findings came up short to establish what type of specific training component enhances effectiveness [138]. The evidence-based review of Dargo et al. [138] implies that the time of implementation is the key to the effectiveness of ACL injury prevention programs, rather than specific training components. These findings seem to be in line with the current hypothesis, that the neuro-energetic resources of the proprioceptive system are limited and these resources could be expanded to some extent by neural adaptation in order to serve injury prevention in unaccustomed or strenuous eccentric exercise moments. The authors of this paper are suggesting that the “locus minoris resistentiae” is at the energy-generating capacity of mitochondria of the proprioceptive terminals in the proximal tibia in their current NC-ACL injury model. Accordingly, it appears that the more efficient is the energy-generating capacity of these mitochondria, the higher is the resistance to mechano-energetic lesions at the terminals of these sensory neurons. It is noteworthy that proprioceptive training not only enhances the neuro-energetic capacity and plasticity of the proprioceptive system at the terminals of the peripheral sensory nerves, but likely at spinal and supraspinal levels as well [30,139,140,141]. It is important to emphasize the findings of Dargo et al. [138] again that more training or more added components to the programs were not leading to better outcome. This could be indicative that the neuro-energetic resources of the proprioceptive system are limited, therefore the neuronal adaptation capacity basis of the proprioceptive training is also capped.

After all it should not be a surprise, that proprioceptive training programs are also appear to be an evidence-based prophylactic measure to decrease the incidence of ankle sprains among athletes. These prevention programs are effective in the presence of ankle sprain history or in the absence of it as well [142].

Further research should be the focus of the component types of neuromuscular and proprioceptive training programs, the duration and the time span of them in order to enhance effectiveness. For example, while static stretching has not been proven to be an effective strategy of preventing general musculoskeletal athletic injuries, but meta-regression analysis seems to substantiate that static stretching could be an effective component within an ACL prevention program [143]. Accordingly, the current NC-ACL injury theory entails an altered and enhanced static encoding of the stretch reflex.

## 7. Conclusions

The current hypothesis suggests that the initial cause of NC-ACL injury could be an acute microdamaging compression injury of the proprioceptive sensory axons with concomitant micro- or stress fracture in the periosteum, epiphysis or/and the subchondral region of the proximal tibia. The authors of this paper further propose a similar dichotomous damage mechanism in NC-ACL injury, like in DOMS and in vertebral compression fracture. This dichotomous injury mechanism comprises a primary sensory axonopathy and a secondary, even harsher tissue damage that includes the injury of the ACL. The primary damage could happen in the repetitive unaccustomed or strenuous accelerating and decelerating exercise moments preceding the actual NC-ACL injury moment. The acute compression or even crush axonopathy of the fastest conducting sensory neurons is suggested to be the primary damage. As a result, the impaired proprioception will lead to the injury of the ACL in the secondary damage phase in a decelerating moment that is under an ASR. The cornerstones of our hypothesis are as follows:

- NC-ACL injury is proposed to be a dichotomous injury mechanism;
- Primary damage could be an acute compression proprioceptive sensory axonopathy in the proximal tibia with concomitant microcracks in the periosteum;
- Secondary damage is a harsher tissue damage when the ACL is also injured, leading to a subluxated knee joint, to bone bruises and to other tissue damage;
- NC-ACL injury is suggested to happen under an ASR in unaccustomed or strenuous eccentric exercise moments;
- Elevated PGE2 and NO are proposed to play a critical role in the initial axonal microdamage signaling in a dose dependent manner;
- A critical mechanism in the central nervous system is proposed to occur in the spinal dorsal horn;
- Activated NMDA receptors under an osteocalcin induced ASR are proposed to play a significant role in modulating the spinal sensory input and in the development of injury, especially the longitudinal aspect of it;
- Delayed latency of MLR is suggested to be indicative of proprioceptive impairment and could be translated as some of the monosynaptic neuronal connections of the stretch reflex are switched to polysynaptic ones;
- LH induced substantial TrkA and NGF gene expression and PGE2 release could explain why non-contact ACL injury is at least three-times more prevalent among female athletes;
- Analog dichotomous injury mechanism and impaired proprioceptive signaling is proposed in delayed onset muscle soreness, compression vertebral fracture and in other non-contact injuries.

Based on this hypothesis, it seems to be the right strategy that recent prevention programs are focusing on neuromuscular control and proprioceptive training, because the proprioceptive sensory capacity could be enhanced with neuronal adaptation, which could serve NC-ACL injury prevention to some extent.
